# Toward high-throughput oligomer detection and classification for early-stage aggregation of amyloidogenic protein

**DOI:** 10.3389/fchem.2022.967882

**Published:** 2022-08-30

**Authors:** Bogachan Tahirbegi, Alastair J. Magness, Maria Elena Piersimoni, Xiangyu Teng, James Hooper, Yuan Guo, Thomas Knöpfel, Keith R. Willison, David R. Klug, Liming Ying

**Affiliations:** ^1^ Department of Chemistry, Imperial College London, London, United Kingdom; ^2^ National Heart and Lung Institute, Imperial College London, London, United Kingdom; ^3^ School of Food Science and Nutrition and Astbury Centre for Structural Molecular Biology, University of Leeds, Leeds, United Kingdom; ^4^ Department of Brain Sciences, Imperial College London, London, United Kingdom

**Keywords:** single-molecule photobleaching, fluorescence imaging, machine learning, artificial neural network, amyloid-β, α-synuclein, protein aggregation, neurodegenerative disease

## Abstract

Aggregation kinetics of proteins and peptides have been studied extensively due to their significance in many human diseases, including neurodegenerative disorders, and the roles they play in some key physiological processes. However, most of these studies have been performed as bulk measurements using Thioflavin T or other fluorescence turn-on reagents as indicators of fibrillization. Such techniques are highly successful in making inferences about the nucleation and growth mechanism of fibrils, yet cannot directly measure assembly reactions at low protein concentrations which is the case for amyloid-β (Aβ) peptide under physiological conditions. In particular, the evolution from monomer to low-order oligomer in early stages of aggregation cannot be detected. Single-molecule methods allow direct access to such fundamental information. We developed a high-throughput protocol for single-molecule photobleaching experiments using an automated fluorescence microscope. Stepwise photobleaching analysis of the time profiles of individual foci allowed us to determine stoichiometry of protein oligomers and probe protein aggregation kinetics. Furthermore, we investigated the potential application of supervised machine learning with support vector machines (SVMs) as well as multilayer perceptron (MLP) artificial neural networks to classify bleaching traces into stoichiometric categories based on an ensemble of measurable quantities derivable from individual traces. Both SVM and MLP models achieved a comparable accuracy of more than 80% against simulated traces up to 19-mer, although MLP offered considerable speed advantages, thus making it suitable for application to high-throughput experimental data. We used our high-throughput method to study the aggregation of Aβ_40_ in the presence of metal ions and the aggregation of α-synuclein in the presence of gold nanoparticles.

## Introduction

Interest in protein misfolding and aggregation in general, and amyloidogenesis in particular, has exploded in the past dozen years, as scientists started to recognize the role of protein aggregates in a number of neurodegenerative diseases as well as other human diseases (Dobson, 2003; Iadanza et al., 2018; Ke et al., 2020). However, some amyloids are actually “functional,” biologically useful, and have been selected for performing important biological tasks, such as the establishment of long-term memory and biofilm formation (Ulamec and Radford, 2020). Arguably, amyloid-β peptide (Aβ) has received the most attention because of its importance in Alzheimer’s disease pathology (Selkoe and Hardy, 2016). High-order Aβ aggregates (“plaques”) are one of the key hallmarks of Alzheimer’s disease (AD), but in fact the most cytotoxic species are low-order oligomers (Benilova et al., 2012). However, the composition of low-order Aβ oligomers is poorly described, and the kinetics of early-stage Aβ aggregation is essentially unknown.

Conventional techniques such as the Thioflavin T (ThT) fluorescence assay (Xue et al., 2017), circular dichroism (CD) spectroscopy (Zhang et al., 2018), nuclear magnetic resonance (NMR) (Suzuki et al., 2013), transmission electron microscopy (TEM) (Eisenberg and Sawaya, 2017), and atomic force microscopy (AFM) (Smith et al., 2006) have all been used to provide ensemble kinetics of amyloidogenic protein aggregation. They can resolve the intermediate species populated during their formation to some degree and the morphology of amyloid fibrils. In the ThT assay, Aβ aggregation kinetic measurements are typically performed at an Aβ concentration range between 1 and 100 μM, several orders larger than its physiological concentration. Kinetic modeling of these aggregation data sets is highly successful in making inferences about the nucleation process and the growth mechanism of fibrils and microscopic kinetic parameters can be characterized (Meisl et al., 2018). However, the evolution from monomer to low-order oligomer in the early stage of aggregation cannot be easily detected by the ThT assay or any other ensemble assays due to the low population of the oligomers and their transient nature. Single-molecule approaches are well suited to detect different molecular species in a complex mixture. A single-molecule method termed two color coincidence detection (TCCD) was reported to be capable of detecting the association of biomolecules at concentrations at low fM concentrations (Orte et al., 2006), and was then adapted to probe the assembly of oligomeric species formed during the aggregation of PI3 kinase (Orte et al., 2008). Single-molecule fluorescence resonance energy transfer (smFRET) has also been proven a powerful method to resolve oligomeric subpopulations during aggregation, and oligomers formed during the aggregation of Yeast Prion Protein Ure2 can be quantified and different types of α-synuclein oligomers can be distinguished (Cremades et al., 2012; Fusco et al., 2017; Yang et al., 2018).

Single-molecule fluorescence loss due to photobleaching results in a stepwise intensity drop for individual fluorophores in a biomolecular complex. Therefore, direct measurement of the number of fluorophore-tagged monomers present in a biomolecular assembly can be achieved by counting the total number of photobleaching steps in the fluorescence intensity profile over time (Leake et al., 2006; Lenn et al., 2011). This tool is well suited to determine stoichiometry of protein complex in living cells (Mehta et al., 2013; Hummert et al., 2021) as well as detect amyloidogenic protein oligomers (Dresser et al., 2021), although sometimes it is hampered by fast photobleaching, which limits the total number of photons that can be detected thereby giving a short time profile for analysis, or photo-induced blinking of the fluorophore (Dickson et al., 1997).

We developed a high-throughput protocol for single-molecule photobleaching experiments under the total internal reflection fluorescence (TIRF) mode with an automated optical microscope and applied it to investigate the aggregation of Aβ_40_ in the presence of metal ions and the aggregation of α-synuclein in the presence of gold nanoparticles. Stepwise photobleaching of dye-labeled Aβ_40_ immobilized onto a solid substrate allowed us to determine stoichiometry of Aβ_40_ oligomers and probe their evolution over time.

It is well documented that Aβ_40_ and Aβ_42_ peptides have differences in their aggregation behavior (Qiu et al., 2015). Aβ_42_ has a higher propensity to aggregate and form protofibrils than Aβ_40_. We aimed to keep the Aβ peptide in its monomeric form as much as possible prior to the start of the aggregation process, therefore we chose Aβ_40_ as our model system in this study.

Copper was found to inhibit the growth of the fibrillar form of Aβ by stabilizing off-pathway prefibrillar Aβ (Pedersen et al., 2011), and the disruption of copper homeostasis plays a crucial role in neurodegeneration (Giampietro et al., 2018). We have previously studied kinetics of copper binding to various Aβ peptides and found that the rate of copper-bridged dimer formation of Aβ carrying the FAD mutation correlates with the onset age of FAD and that the timescale of the redox cycling of Aβ-Cu complex is biologically relevant (Branch et al., 2015; Girvan et al., 2016; Branch et al., 2017; Girvan et al., 2018). We, therefore, applied the high-throughput method to address the influence of copper on the Aβ oligomer distribution as a function of time under sub-micromolar Aβ concentration, a case closer to a physiological condition, yet intractable by a conventional method.

α-Synuclein is a 140 residue intrinsically disordered protein that is highly enriched in presynaptic terminals and its aggregation is associated with Lewy body dementias (LBDs) including Parkinson’s disease (PD) (Goedert, 2001). Oxidative stress plays an important role in the degeneration of dopaminergic neurons in PD (Dias et al., 2013). Recently we demonstrated that α-synuclein coated gold nanoparticles were able to induce oxidative stress in neuroblastoma cells which could then be rescued by antioxidant lipoic acid-coated gold nanoparticles (Piersimoni et al., 2020). Herein we showed that our high-throughput approach can differentiate oligomer distributions at the same time point on aggregation curves under different nanoparticle concentrations.

Furthermore, we have investigated the potential application of supervised machine learning to classify photobleaching traces into stoichiometric categories based on an ensemble of measurable quantities of the traces themselves. Both traditional machine learning using support vector machines (SVMs) (Huang et al., 2018) and a neural network model based on multilayer perceptron (MLP) (Yu et al., 2019) were found to achieve on average more than 80% accuracy against simulated traces, yet the later runs much faster and hence better suited for high-throughput applications.

## Results

**FIGURE 1:**
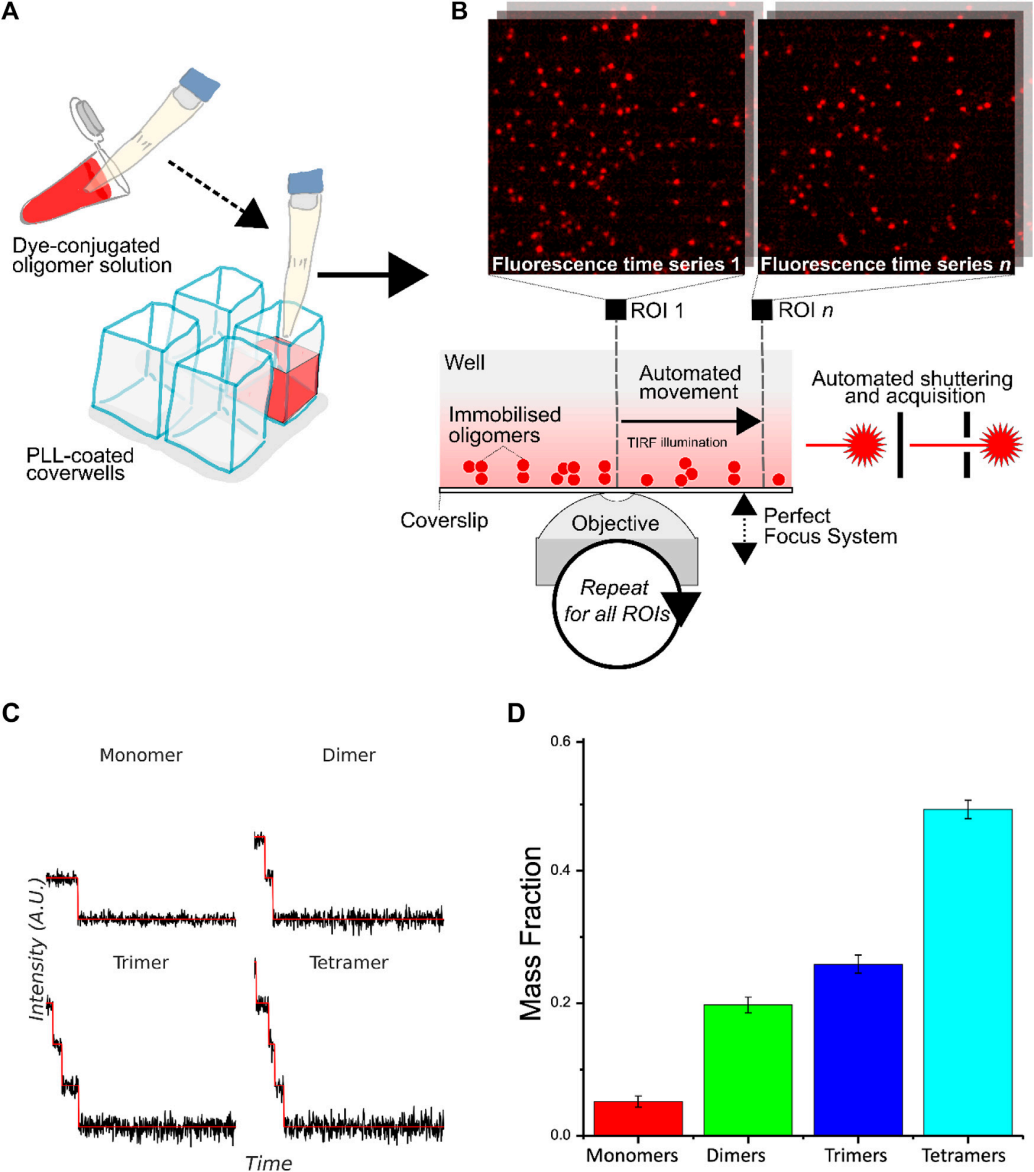
Workflow of high-throughput protein oligomer analysis by single-molecule stepwise photobleaching. **(A)** Dye-labeled protein sample is immobilized onto a solid-phase substrate or lipid membrane inside cover glass-bottomed chambers. **(B)** Overview of the automated acquisition process. An automated piezoelectric stage is used to rapidly acquire large numbers of photobleaching traces over preset regions of interest (ROIs) in the glass well. Immobilized oligomers are viewed with TIRF illumination with focus maintained over large distances with a computer-controlled focusing system, with each fluorescence time series acquired automatically via custom control software automating laser shuttering and image acquisition. Example images of immobilized oligomers from two different ROIs are shown. **(C)** Illustrative idealized photobleaching traces for a monomer, dimer, trimer, and tetramer. **(D)** Normalized distribution of the number of fluorophores in CD209 tetramers determined experimentally (N ∼ 6000).

We designed and programmed a TIRF imaging microscope with an autofocus system to collect precise, reliable, and clear photobleaching data. We used customized analysis code written in FIJI and MATLAB to process raw image sequences and extract time-dependent intensity traces of all single molecules that can be identified. We then used the Progressive Idealization and Filtering (PIF) software developed by Blunck and coworkers (McGuire et al., 2012) to determine the stoichiometry of oligomers from photobleaching time profiles. The combination of two codes enabled us to analyze hundreds of molecules simultaneously for a given image sequence, allowing high-throughput and excellent statistics on experimental datasets. Figures 1A–C briefly show the workflow of our high-throughput single-molecule photobleaching method. First, the sample at a given aggregation time point was diluted and single molecules (monomers and oligomers) were immobilized on polylysine coated surface of glass wells (Figure 1A). TIRF imaging of single molecules was then carried out on each glass well using an automated optical microscope equipped with a Perfect Focusing System (Figure 1B). Single-molecule photobleaching traces were obtained and then classified according to their number of photobleaching steps determined by PIF (Figure 1C). Large datasets can be analyzed without user bias in the selection or interpretation of the data. With such automatized data collection and analysis, we can determine stoichiometry of a hundred thousand oligomers in a day.

We first tested the accuracy and robustness of the method by CD209 (also known as DC-SIGN). CD209 is a C-type lectin receptor of dendritic cells involved in early stages of numerous infectious diseases. It is naturally organized into a tetramer, thereby enabling multivalent interaction with pathogens (Feinberg et al., 2005). The protein was site-specifically labeled by ATTO 488 dye with an efficiency of ∼75 % determined by mass spectrometry. Since not all protein monomers were labeled by a fluorophore, we predicted the probability *p* of detecting the number of fluorophores *n* in a tetramer by the following equation: $p_{n}=\frac{4!}{n!\left(4-n\right)!}\gamma^{n}{\left(1-\gamma \right)}^{4-n}$ , where γ is the labeling efficiency. Based on our result as shown in Figure 1D, we estimated the labeling efficiency to be around 80%, in good agreement with the experimentally measured value, confirming that the protein was predominantly in its tetrameric form. Given that pre-photobleaching before imaging acquisition was negligible in our experiment, we concluded that apparent monomers, dimers, and trimers observed were not only due to insufficient labeling but also due to their dissociation after dilution to pM concentration for single-molecule imaging.

**FIGURE 2:**
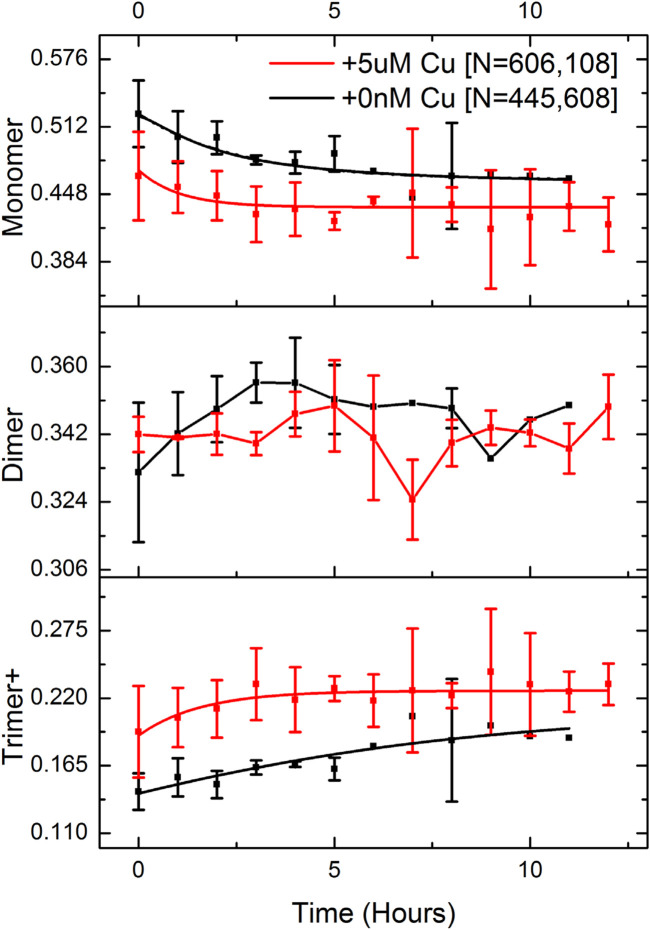
Kinetic profiles of Aβ aggregation are determined by single-molecule photobleaching analysis. 500 nM Aβ was incubated in the presence and absence of 5 µM Cu^2+^. Mass fraction instead of molar fraction in the *Y*-axis was used to better represent the relative population of oligomers. Oligomers with sizes larger than trimer were binned together with trimers and shown as “Trimer+“. Three experimental repeats were carried out for each condition (+/- copper) and over 1 million single-molecule photobleaching traces were analyzed in total.

We then attempted to probe kinetics of the aggregation of Aβ_40_ below µM concentration. We studied Aβ_40_ oligomerization under a varied set of near-physiological conditions with single-molecule photobleaching techniques in the presence of copper. Various immobilization strategies for Aβ_40_ were explored, including absorption onto the unmodified borosilicate glass surface, immobilized onto a poly-l-lysine (PLL) substrate, and insertion into supported phospholipid bilayers. We found that PLL was the best choice for the immobilization of Aβ_40_ oligomers on the surface. Fluorescent foci were analyed by measuring the integrated intensity throughout 750 frames, and stoichiometry of photobleaching traces was determined via PIF (McGuire et al., 2012). We acquired kinetic oligomerization profiles over a period of 12 h with and without copper. 500 nMAβ_40_ was incubated with and without 5 µM Cu^2+^ and a minute volume of sample at each time point were taken and diluted to 50 pM before being applied to a PLL-coated glass chamber. The low Aβ/Cu stoichiometry case better resembles the physiological condition where low nM Aβ are present, while copper released to the synaptic cleft could reach µM concentrations (Branch et al., 2015). Figure 2 shows the time profiles determined for the Aβ_40_ monomer, dimer, trimer, and above. The mass fraction was used to better represent the amount of Aβ_40_ in different oligomeric states. Unlike most amyloidogenic protein aggregation kinetic experiments that have been reported, the single-molecule method facilitates direct access to the distribution of different sized oligomers as a function of time, making detailed kinetic analysis of the initial stage of aggregation feasible.

We found the presence of a significant amount of pre-formed Aβ_40_ oligomers when samples were incubated at 500 nM concentration (time = 0). More trimers and larger oligomers (trimer+) were detected in the presence of copper even for the time 0 measurement. Interestingly, while mass fractions of monomer and trimer+ exhibit either a time-dependent decay or rise, the dimer fraction fluctuates around a constant mean, suggesting that the dimer could be the key intermediate in pre-equilibrium with the monomer and other oligomers on the pathway to nucleation. However, this result does indicate that there is no dramatic effect of copper on the timescale of oligomer formation in Aβ_40_ aggregation at this stage.

Next, we investigated the potential mechanism responsible for the delayed fibrilization of α-synuclein in the presence of an increasing concentration of gold nanoparticles, by monitoring the distribution of α-synuclein oligomeric species in the single-molecule regime.

**FIGURE 3:**
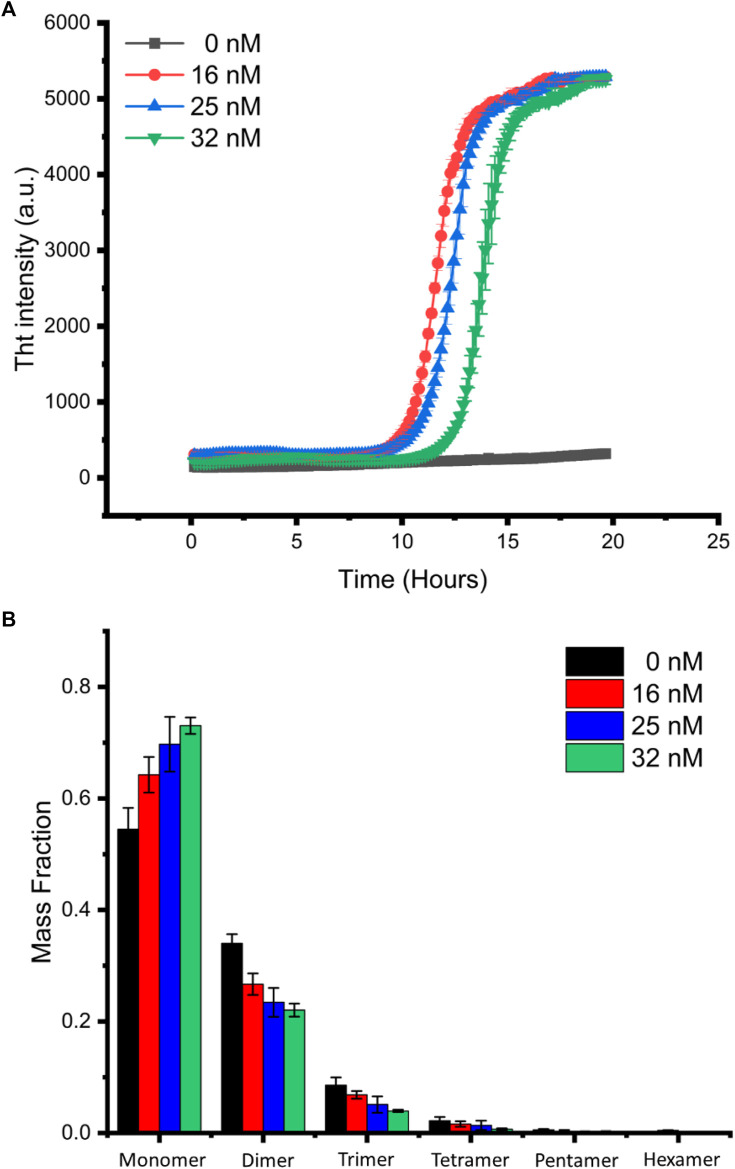
Single-molecule analysis of α-synuclein oligomer distribution. **(A)** α-Synuclein aggregation in the presence of different concentrations of gold nanoparticles was monitored by ThT assay. The curves shown are the average of the triplicates. **(B)** Mass fraction of α-synuclein species in aggregation solution at 12-h incubation.

Figure 3A shows the ThT assay result for the aggregation of α-synuclein at different gold nanoparticle concentrations, suggesting that gold nanoparticles promoted protein aggregation but delayed the growth phase when its concentration increased. We labeled α-synuclein with alexa 647 at position 7 which was mutated from glycine to cysteine for single-molecule detection. Single-molecule analysis of the distribution of α-synuclein species for samples taken at 12 h after mixing 20 µM labeled α-synuclein with 16, 25, and 32 nM 20 nm diameter gold nanoparticles, results shown in Figure 3B, clearly indicates the increase of monomer fraction and the decrease of oligomer fractions as the concentration of gold nanoparticles increases. Since single-molecule data were acquired at the time point located at the growth phase of the aggregation (Figure 3A), this result correlates well with the ThT assay which reveals the order of increased degree of aggregation is the reverse order of the nanoparticle concentration. We note that, under our experimental condition, aggregation of α-synuclein in the absence of gold nanoparticles was not observed by the ThT assay (Figure 3A), yet oligomers were relatively more populated in this sample than all others as revealed by single-molecule analysis. It is likely that, on one hand, the binding of α-synuclein to gold nanoparticles can enhance seed formation, thus promoting aggregation; on the other hand, when the ratio of nanoparticle to α-synuclein increases, oligomers can also be sequestered by excess binding surface available on gold nanoparticles, thus delaying aggregation.

We have demonstrated the potential of the high-throughput single-molecule photobleaching in obtaining oligomeric species distribution in amyloidogenic protein aggregation. However, the PIF (McGuire et al., 2012) algorithm is computationally costly, with computing times becoming prohibitive for very large numbers of analyzed molecules, thus precluding real-time analysis of distributions during high-throughput data acquisition.

To address this data analysis bottleneck, we started to investigate an alternative solution to the problem of step identification in photobleaching traces. We used supervised machine learning to classify bleaching traces into stoichiometric categories based on an ensemble of measurable quantities of traces themselves. We used machine learning techniques to learn partitions of this feature space according to the number of monomers comprising each simulated trace and thus assign stoichiometry based on learned features of the dataset.

**FIGURE 4:**
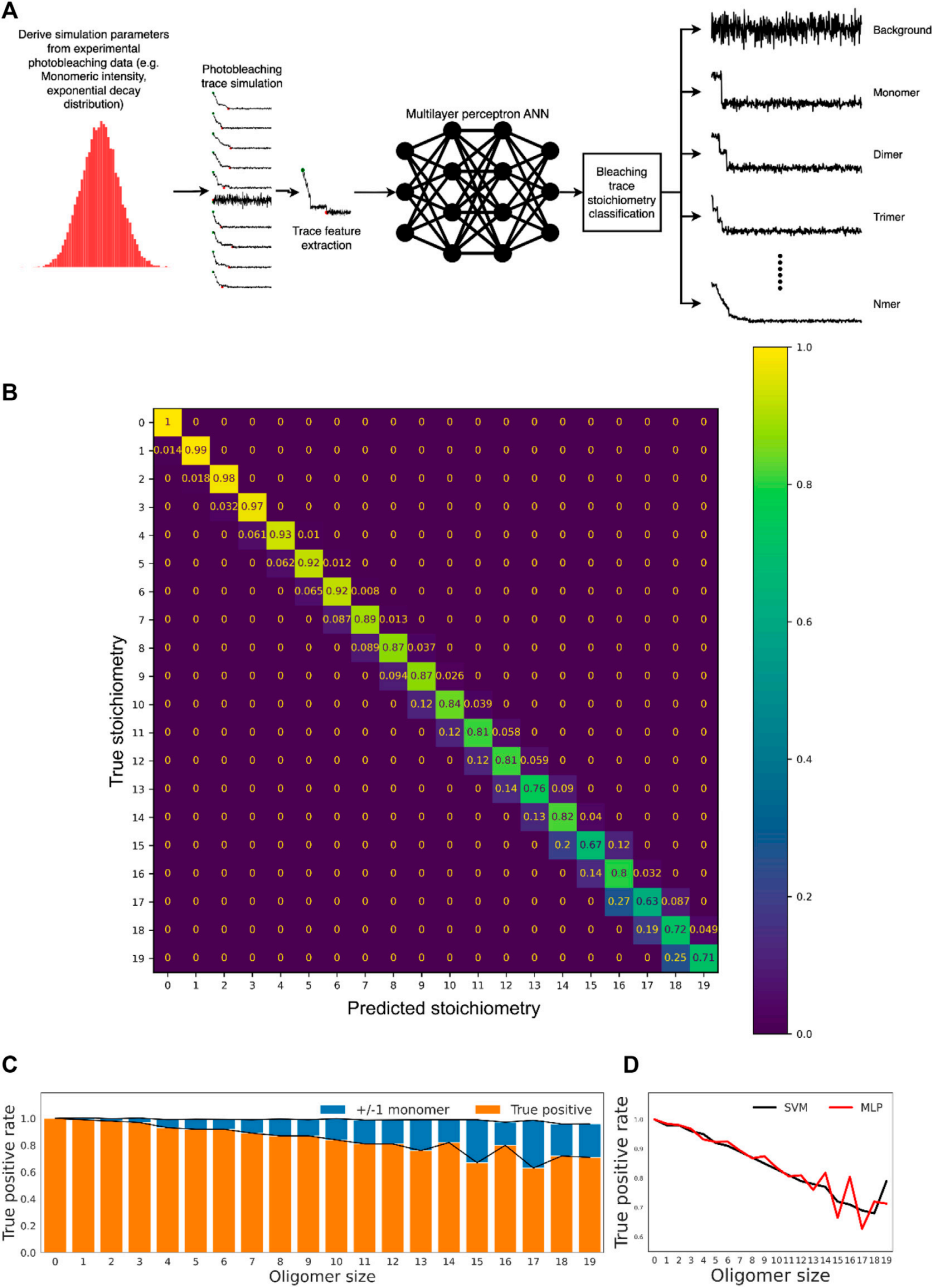
Machine learning-based classification of realistic photobleaching traces allows accurate determination of oligomer stoichiometry. **(A)** Overview of the computational workflow employed for oligomer subunit prediction with an MLP model. **(B)** Confusion matrix showing performance of the MLP model on the held-out test data. The scale indicates the fraction of each ground truth stoichiometric class predicted to have a given stoichiometry. **(C)** True positive rates for the model across all stoichiometric classes. “Relaxed” true positive rates are also shown, where classification is considered correct if the classification is only ± 1 monomeric unit from the true value. The average relaxed true positive rate across all classes is 98.9 ± 1.1%. **(D)** Classification accuracy of MLP and SVM models was comparable across all oligomer classes, with both approaches displaying a drop-off in accuracy for higher-order oligomers.

Simulated traces were constructed first due to the lack of accurate “ground truth” classifications for experimental photobleaching traces as well as to compensate for the relative lack of traces for higher oligomers since without balanced training data for each oligomer type the model would become biased towards classes for which it has seen the most examples. Simulated traces were produced by creating a 500-frame step function of initial intensity that corresponds to a random sampling of the single-step photobleaching intensity distribution from the single-molecule data. Gaussian noise was added to simulate microscopy imaging noise, with parameters set matching those measured experimentally from images. Multimeric molecules were produced as the linear sum of two or more such traces. Supervised classification learning was conducted using multiple models, with the most accurate determined to be either a two-layer multilayer perceptron neural network (Yu et al., 2019) or an RBF-kernel support vector machine (Huang et al., 2018). Machine learning trials with simulated photobleaching traces up to 19-mer were carried out. Figure 4A shows the workflow of the machine learning and classification process.

As shown in the confusion matrix (Figure 4B), misclassification of oligomer stoichiometry was generally limited to ± 1 monomeric unit from the true value (see Figure 4C for more details), and the overall accuracy is 83.5%. In general, the prediction accuracy decreases with the increase of oligomer size, and the multilayer perceptron (MLP) neural network and the RBF-kernel support vector machine (SVM) performed equally well (Figure 4D and Supplementary Figure S2). Furthermore, the method avoids extreme misclassification of complex higher-order traces though only the performance up to 19-mer was investigated. Taken together, the approach is a viable and accurate strategy for oligomer stoichiometry counting, with obvious utility in single-molecule aggregation kinetics measurements.

## Discussion

We established a high-throughput method to monitor the progression of early-stage protein aggregation based on the analysis of single-molecule photobleaching profiles. The method enabled us to examine the time course of the population of small Aβ_40_ oligomers, indicating the feasibility of directly probing aggregation kinetics of Aβ_40_ at the level of individual oligomeric species. Subtle shifts in populations of oligomer species could be observed under near-physiological aggregation conditions.

As a demonstration of the method, we investigated the influence of copper on Aβ_40_ aggregation in the induction phase which would be normally masked by studies using traditional measurements such as the ThT assay. Our previous study (Branch et al., 2015; Branch et al., 2017) indicated that the binding of Cu^2+^ to Aβ is near diffusion limited and the binding would be completed within milliseconds in the synaptic cleft as well as under our experimental condition. Therefore, even at the first time point of the measurement (t = 0), each Aβ molecule was bound to a Cu^2+^ ion, resulting in a decrease in the brightness of the fluorophore labeled at the N-terminus of Aβ by about ∼60% (Branch et al., 2015). However, our imaging platform and data analysis tool performed well when facing such a reduction in the fluorescence photon count rate. Admittedly, we were unable to start the reaction at 100% Aβ_40_ monomer even though significant efforts were made in refining the sample preparation because Aβ_40_ is a high-aggregation-prone peptide, and as a result, it is difficult to obtain a peptide that is free of pre-aggregates. This is a major issue well recognized in the Aβ aggregation research (Bharadwaj et al., 2009). At 500 nM, significant populations of Aβ_40_ dimer and other small Aβ_40_ oligomers were present, which hampered detailed mechanistic modeling of the experimental data, even though evolutions of the relative population of Aβ_40_ monomer and small oligomers were obtained. Our attempt with a low pM concentration sample suggests that *in situ* detection of the time profile of dimers and small oligomers is possible when a low pM sample is monitored, yet the continuous build-up of molecules on the surface would eventually pose a limit to how long the aggregation process could be monitored (Supplementary Figure S1). Further optimization of the sample and sample immobilization will be necessary. One option would be the use of recombinant Aβ, which has been shown essential to obtaining high-quality aggregation data in the ThT assay (O’Malley et al., 2018). On the sample capturing side, a set of antibodies which can recognize different Aβ species from monomer to small oligomers is desirable for our single-molecule detection platform. Encouragingly, progress has been made recently in the rational design of a conformation-specific antibody for the quantification of Aβ oligomers (Aprile et al., 2020).

Using single-molecule photobleaching analysis, we successfully differentiated the monomer and small oligomers present and their relative populations in the growth phase of α-synuclein aggregation promoted by 20 nm gold nanoparticles. Our results regarding the concentration-dependent effect of gold nanoparticles on the half-life of the aggregation process differ from the literature (Álvarez et al., 2013), exhibiting an increase rather than a reduction upon the increase of gold nanoparticle concentration. Since our single-molecule result agrees with that of the ThT assay, we attribute this discrepancy to disparities in the fluorescent indicator used and the protein/gold nanoparticle ratio in these two studies. The aggregation assay in the literature used a small fraction of dye-labeled protein called MFC which is considerably more sensitive in the detection of the early stages of the aggregation than ThT (Yushchenko et al., 2010). Because of the lower protein concentration used in our study (20 vs. 100 µM), it is likely that at a low protein/gold nanoparticle ratio, oligomeric protein would be sequestered and stay on gold nanoparticles, hence reducing the number of oligomers available for seed formation in the induction phase. Consequently, a prolonged induction phase was observed in our study when the concentration of gold nanoparticles increased.

Single-molecule approaches have the potential to underpin the working principles of complex biomolecules as well as reveal kinetic mechanisms of the protein aggregation process. However, such approaches generally require extensive sampling to obtain statistically robust data on distributions of states and kinetic rate constants connecting these states. Although emerging experimental techniques, such as the one we used here, can generate large datasets, existing analysis tools are not suitable to process the large volume of data obtained in the high-throughput mode, especially when on-the-fly analysis and display of the result are preferable. We note that the well-known Chung-Kennedy algorithm (Chung and Kennedy, 1991) and the Hidden Markov model (HMM) analysis (Messina et al., 2006) are computationally expensive. The application of machine learning approaches in single-molecule analysis has gained considerable interest recently (Meng et al., 2022; Xu et al., 2019; Yuan et al., 2020; Thomsen et al., 2020; White et al., 2020; Li et al., 2020). A deep learning algorithm named DeepFRET has been demonstrated to reach classification accuracy on ground truth data by over 95%, not only outperforming human operators but also reducing the computation time by two orders of magnitude (Thomsen et al., 2020). Another unsupervised machine learning algorithm called DISC can accelerate the analysis of single-molecule trajectories by three orders of magnitude with improved accuracy in comparison to commonly used algorithms (White et al., 2020). Most notably, a convolutional and long-short-term memory deep learning neural network (CLDNN) used by Xu and co-workers (Xu et al., 2019) achieved much higher accuracy and two orders of magnitude improvement in efficiency compared to HMM for small oligomers up to tetramer. Built upon the success of CLDNN, an unsupervised learning framework of the discriminator-generator network (DGN) was developed which outperforms both HMM and PIF (Yuan et al., 2020). We adopted supervised learning approaches based on lightweight artificial neural networks (Yu et al., 2019) as well as RBF-kernel support vector machines (Huang et al., 2018), and these approaches demonstrated excellent capability to classify oligomers up to 19-mer, well beyond the capability of trace idealization algorithms (PIF, HMM etc.). Classification time for a given trace was on a sub-millisecond timescale, opening the door to real-time analysis of oligomer distributions during high-throughput experiments. In comparison to the literature study (Xu et al., 2019; Yuan et al., 2020), the accuracy of our deep learning methods is similar, but we have demonstrated that our approach is applicable to even larger oligomers. Furthermore, our approach is quite flexible since photobleaching and imaging noise are physical phenomena well understood, making simulation a valid way to train a model and obtain training parameters before applying them to experimental photobleaching traces. Future study to compare the accuracy and efficiency of different machine learning algorithms is desirable to establish a unified approach for single-molecule photon trajectory analysis.

However, in comparison to label-free detection methods which would generally not perturb the aggregation system under investigation, our high-throughput method does require site-specific fluorescent labeling which might alter the physicochemical property of the protein, thereby influencing the aggregation process. The effect of dye labeling on Aβ aggregation has recently been investigated in detail (Wägele et al., 2019), indicating that HiLyte 647 label tends to favor the formation of large oligomers in comparison to the HiLyte 488 label though standard ThT assays used to investigate the kinetics of β-sheet formation display no delay for HiLyte 647-Aβ_40_ compared to wild-type Aβ_40_. It was concluded that for HiLyte 647-Aβ_40_, not the nucleation but the fibril growth was modified. In the case of α-synuclein aggregation, dye labeling is generally nonperturbative as illustrated by a combination of *in vitro* aggregation kinetics measurements and imaging of the resulting fibrils (Haney et al., 2016). However, it can have a pronounced effect on the morphology of α-synuclein fibrils (Mučibabić et al., 2016).

Another notable limitation is that our methodology requires surface immobilization of the sample for single-molecule imaging, during which protein oligomers and monomers might further assemble on the surface to form larger oligomers. On the other hand, dilution of sample concentrations well below nM would potentially induce the dissociation of oligomers prior to their immobilization. Therefore, care should be taken when comparing results obtained from high-throughput single-molecule analysis with label-free ensemble measurements.

Nevertheless, the high-throughput method is capable of screening compounds that can inhibit protein aggregation. We are aware that the use of chemical kinetics has recently enabled accurate quantifications of microscopic aggregation steps that lead to the proliferation of protein oligomers and the kinetics strategy has been successfully used to screen small molecules inhibiting oligomer formation in α-synuclein aggregation (Staats et al., 2020). Since our platform can directly measure the time profile of oligomer distribution, we expect that it can be readily used to determine the effect of compounds that prevent or reduce amyloid oligomer formation.

In summary, we have introduced our recent efforts in developing a high-throughput method for amyloidogenic protein oligomer classification based on single-molecule stepwise photobleaching analysis. Applications of our method in copper-induced early stage Aβ_40_ aggregation as well as in gold nanoparticle-promoted α-synuclein aggregation have demonstrated, to some extent, the potential of such method in elucidating the aggregation mechanism of Aβ under physiological conditions and facilitating the understanding of the possible role of copper in the initiation of Aβ aggregation in the synaptic cleft which is inaccessible by experimental tools currently available. Furthermore, we have demonstrated the successful application of supervised deep learning approaches to classify single-molecule photobleaching traces and determine the oligomer size. We envisage that artificial intelligence is coming to age in single-molecule studies of amyloidogenic protein aggregation linked to neurodegenerative diseases.

## Materials and methods

### Aβ_40_ sample preparation

HiLyte™ Fluor 647-labeled Aβ_40_ (HPLC purity >95%) was purchased from Anaspec. Lab-Tek Chambered 1.0 Borosilicate cover glass was purchased from Thermo Fisher Scientific. Aβ_40_ powder was dissolved in a HFIP (1,1,1,3,3,3-Hexafluoro-2-propanol) solution to break preexisting β-sheet structures and vortexed gently to mix well. The solution was divided into aliquots and dried in open tubes overnight in the fume hood. The peptide was then further dried under vacuum for 2 hrs to evaporate HFIP residues and kept at -20°C.

### Expression and dye labeling of CD209 and α-synuclein

An extracellular segment of CD209 (also known as DC-SIGN) was constructed as described (Mitchell et al., 2001). Glutamine at position 274 was mutated to cysteine which was labeled by ATTO 643. α-Syn was expressed using pT7-7 α-Syn WT, a gift from Hilal Lashuel (Addgene plasmid # 36,046). A glycine to cysteine mutation was introduced at position 7 and the protein was labeled by Alexa 647 (Details in Supplementary Material).

### Synthesis of gold nanoparticles

The citrate-stabilized gold nanoparticle was synthesized by the sodium citrate method (Frens, 1973). 50 ml of the 1 mM chloroauric acid solution (HAuCl4, cat. no. 484385, Sigma-Aldrich) was heated with stirring until boiling and different volumes of 38.8 mM sodium citrate were rapidly added. The solution was then kept at boiling for further 15 min to give a wine-red solution which was cooled at room temperature with stirring. The hydrodynamic diameter of gold nanoparticles was determined to be 20 nm by DLS using Malvern Zetasizer Nano series (Malvern Instruments, Worcestershire, United Kingdom).

### Thioflavin T assay of α-synuclein aggregation

ThT was diluted in a phosphate-buffered saline (PBS) at the final concentration of 20 μM per well. Various amounts of gold nanoparticles were mixed with α-synuclein and ThT at a final concentration of 20 µM for the protein, 20 µM for ThT, and 16 nM, 25 nM, and 32 nM for the gold nanoparticle. Mixtures were incubated in a 96-well plate at 37°C with shaking every 5 min. After each round of shaking, mixtures were excited at 440 nm and fluorescence emission at 485 nm was recorded.

### Immobilization of Aβ_40_, CD209, and α-synuclein oligomers for single-molecule imaging

A 200 µL 0.01% poly-l-lysine (PLL) solution was added to wells and kept for 5 min before draining slides. The slides were stored overnight at room temperature for the next day’s use. In the case of Aβ_40_, an aliquot was resuspended in the HEPES buffer (50 mM Sodium-HEPES, 100 mM NaCl, pH 7.5) to make the final concentration of the peptide 500 nM. The Aβ_40_ solution was then incubated with and without 5 µM Cu^2+^. A minute sample at each time point was taken and diluted to 50 pM before being applied to PLL-coated glass chamber. In the case of α-synuclein, 20 µM α-synuclein in the HEPES buffer was incubated with different concentrations of gold nanoparticles and a minute sample at 12-h incubation time was taken and diluted to 2 pM before being applied to PLL-coated glass chamber for imaging. Similarly, 20 pM CD209 in the HEPES buffer was applied to PLL-coated glass chamber for imaging.

### Single-molecule total internal reflection fluorescence imaging

A total internal reflection fluorescence (TIRF) microscope based on an inverted Eclipse Ti-E optical microscope (Nikon, Japan) equipped with a 60× NA 1.49 oil immersion objective (Nikon, Japan) and an electron-multiplied CCD (IXON DU-897E, Andor Technologies, Ireland) was used for the acquisition of imaging sequences. Two single-mode fiber coupled diode pumped solid state lasers at 473 nm (Cobolt Blues 25, Sweden) and 647 nm (Laser 2000; United Kingdom) were used as the excitation source. Laser power at the back aperture was adjusted by neutral density filters to 1.5 mW and 0.7 mW for the 473 nm and 647 nm excitation, respectively. Our customized code enabled simultaneous laser exposure and image recording, thus minimizing pre-photobleaching of fluorophores. The code performed automated photobleaching travels in a vertical line downward from the top of the wells. It took approximately 12.5 min to take 20 images of 750 frames with a 40 ms exposure time. Different areas inside each chamber were imaged automatically by an encoded high-speed XY stage with the focal plane being maintained by a Perfect Focus System (PFS) (Nikon, Japan).

### Extraction and analysis of single-molecule photobleaching traces

A customized multi-step image processing and data analysis MATLAB code was developed to facilitate the extraction of photobleaching time profiles and follow-on oligomer classification. Single-molecule photobleaching traces were extracted from raw image sequences via an ImageJ macro, which performed the following image processing operations. Images were first median filtered (radius = 2 pixels) to improve S/N ratios. Oligomers were identified by performing a maximum intensity projection and maxima above a defined threshold were obtained in this image sequence with the ImageJ Find Maxima function. Peak locations were used to define a circular ROI with a 4-pixel radius where counts were integrated for time series to generate photobleaching time traces. Subsequently, PIF software (McGuire et al., 2012) was used to analyze photobleaching time traces.

### Machine learning for oligomer size classification

We investigated artificial neural networks (ANNs) and support vector machines (SVMs) as methods for treating oligomer stoichiometry determination from photobleaching trace measurements as a multiclass classification problem. Both types of models have found widespread application in bioinformatics in recent years (Huang et al., 2018; Yu et al., 2019). We developed bleaching trace simulations in Python which harnessed key experimentally derivable quantities of the fluorophore and imaging system under investigation to produce realistic and experimentally representative traces for oligomers of up to 19 subunits. We simulated 10,000 oligomers for each stoichiometric class, including an additional class for traces where no oligomer was present (i.e., imaging background noise). We used the sklearn library to partition the data into train, validation, and test sets with a ratio of 80:10:10. Training was performed with train and validation sets, with the test set held out for performance assessment after training. Prior to input to the model, the dataset was scaled to zero mean and unit variance. We implemented SVM models using sci-kit learn (Pedregosa et al., 2011) in Python as well as MATLAB, whilst ANN models were written in Python using TensorFlow 2 (https://github.com/tensorflow) and Keras (https://keras.io). Codes on GITHUB are available upon request.

Our MLP neural network model consists two dense, fully connected sequential layers of 256 neurons per layer with the ReLu activation function. Each dense layer is followed by a dropout layer with a dropout rate of 0.5, and the final output layer implemented a softmax activation to convert layer outputs into probability scores that a given trace belongs to each of the stoichiometric class. We trained the model from a random weight initialization with categorical cross-entropy loss, and with a learning rate of 1e-4 using the Adam optimizer. We used a batch size of 256 during training. In contrast to SVM models, the MLP model trained rapidly on the 200,000 simulated trace dataset, in under 30 s on a single NVIDIA Quadro RTX5000 16 GB GPU.

We were able to perform classification with raw trace data (Supplementary Figure S3); however, performance was improved by using a feature vector of derivable parameters which summarizes important facets of the bleaching trace. This simple ensemble of predictors consists the initial trace intensity, the integrated intensity of the entire trace, the trace standard deviation, the trace kurtosis, a traveling window calculating the mean intensity every 20 frames for the first 200 frames, and a feature termed the “bleaching gradient”—the ratio of the initial trace intensity to the final bleaching time. Across all stoichiometries simulated, with the multilayer perceptron (MLP) model, on average, 98.9% of oligomers were classified within ± 1 oligomeric subunit of the ground truth value.

## Data Availability

The original contributions presented in the study are included in the article/Supplementary Material; further inquiries can be directed to the corresponding author.
