# Supplementary material for: Toward high-throughput oligomer detection and classification for early-stage aggregation of amyloidogenic protein
Source: Front Chem. 2022 Aug 30;10:967882. doi: 10.3389/fchem.2022.967882 (PMC9468268; doi:10.3389/fchem.2022.967882)
Supplement: Supplementary file 1 [file DataSheet1.docx]

**Supplementary Material**

**Expression and dye labelling of CD209 and α-synuclein**

Extracellular segment of CD209 (also known as DC-SIGN) was constructed as described (Mitchell et al., 2001). For site specific labelling of the product, glutamine at position 274 was mutated to cysteine (named as CD209Q274C) using standard molecular biology techniques and mutant was confirmed by DNA sequencing. The protein was expressed in E coli and purified by affinity chromatography using a mannose-Sepharose column. Protein labelling was conducted in buffer containing 20 mM HEPES pH 7.2, 150 mM NaCl and 25 mM CaCl_2_. (Guo et al., 2017). CD209Q274C was mixed with maleimide-ATTO 488 with protein to dye ratio of 1:3 at room temperature for 1 hr and then kept at 4 ^o^C for overnight. Labelled protein was then purified using a mannose-Sepharose column and labelling efficiency was determined by high resolution mass spectrometry to be ~ 75%.

α-Syn was expressed in E. coli using pT7-7 α-Syn WT, a gift from Hilal Lashuel (Addgene plasmid # 36046). A glycine to cysteine mutation was introduced at position 7 using a Phusion Site-Directed Mutagenesis Kit (Thermo Fisher Scientific, Massachusetts, USA) for site-specific labelling. The G7C-αSyn was expressed and purified as described previously (Teng 2021). G7C-αSyn was labelled with Alexa Fluor 647 C2 maleimide (Thermo Fisher Scientific, Massachusetts, USA) according to the instruction provided. Briefly, 10 mM dye stock solution was pre-prepared in dimethyl sulfoxide and mixed with disulphide bonds reduced G7C-α-Syn solution to a final molar ratio of 3:1 (dye: protein). The mixture was stirred in the dark for 3 h. Then the mixture was desalted using a PD-10 desalting column containing Sephadex G-25 resin (GE Healthcare Life Sciences, Illinois, USA), and concentrated using 10K MWCO pierce protein concentrators (Thermo Fisher Scientific, Massachusetts, USA) to remove all free dye. The final labelled protein concentration was determined by UV-Vis absorbance and the labelling efficiency was determined to be 95%. The labelled samples were stored at -80 ^o^C.

**References**

Guo, Y., Nehlmeier, I., Poole, E., Sakonsinsiri, C., Hondow, N., Brown, A., Li, Q., Li, S., Whitworth, J., Li, Z., Yu, A., Brydson, R., Turnbull, W.B., Pöhlmann, S., and Zhou, D. (2017). J. Am. Chem. Soc. 139, 11833-11844.

Mitchell, D.A., Fadden, A.J., and Drickamer, K. (2001). A novel mechanism of carbohydrate recognition by the C-type lectins DC-SIGN and DC-SIGNR: subunit organization and binding to multivalent ligands. J. Biol. Chem. 276, 28939-28945.

Teng X., Sheveleva A., Tuna F, Willison K. R., and Ying L. (2021). Acetylation rather than H50Q mutation impacts the kinetics of Cu(II) binding to α-synuclein. ChemPhysChem 22, 2413-2419.


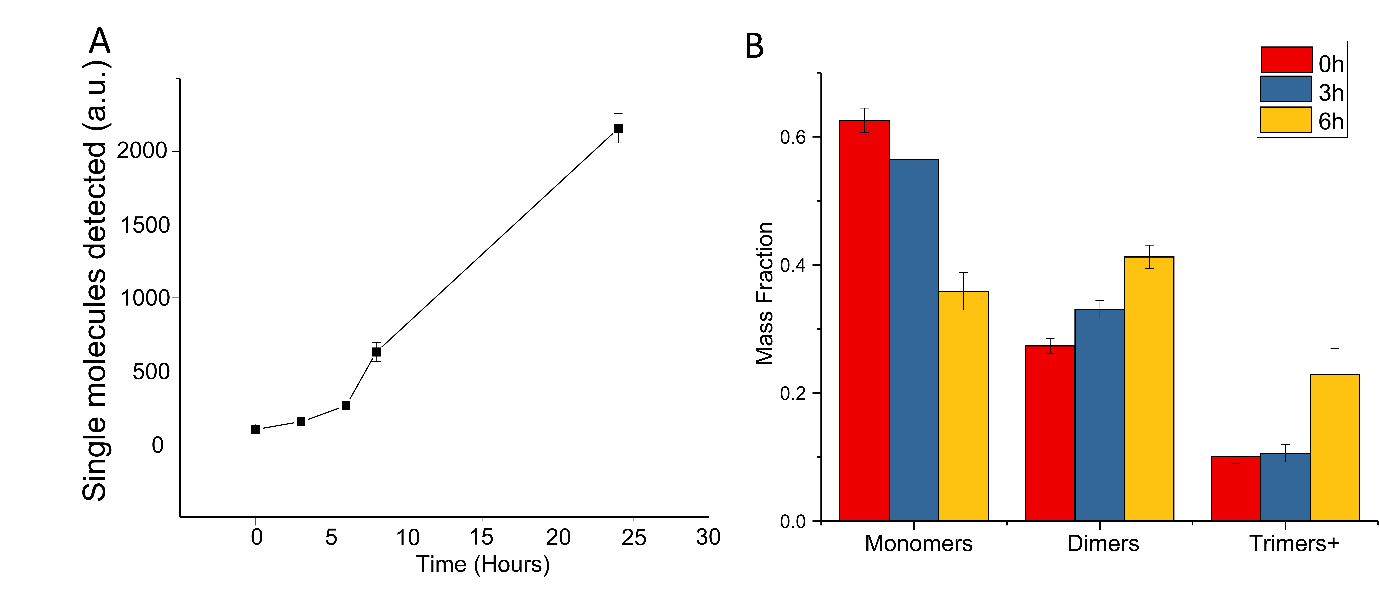


**Figure S1.** Aβ attachment to the surface of glass chamber (A) and single molecule detection of oligomer distribution (B). Experimental condition: Aβ 50 pM, Cu^2+^ 500 nM.


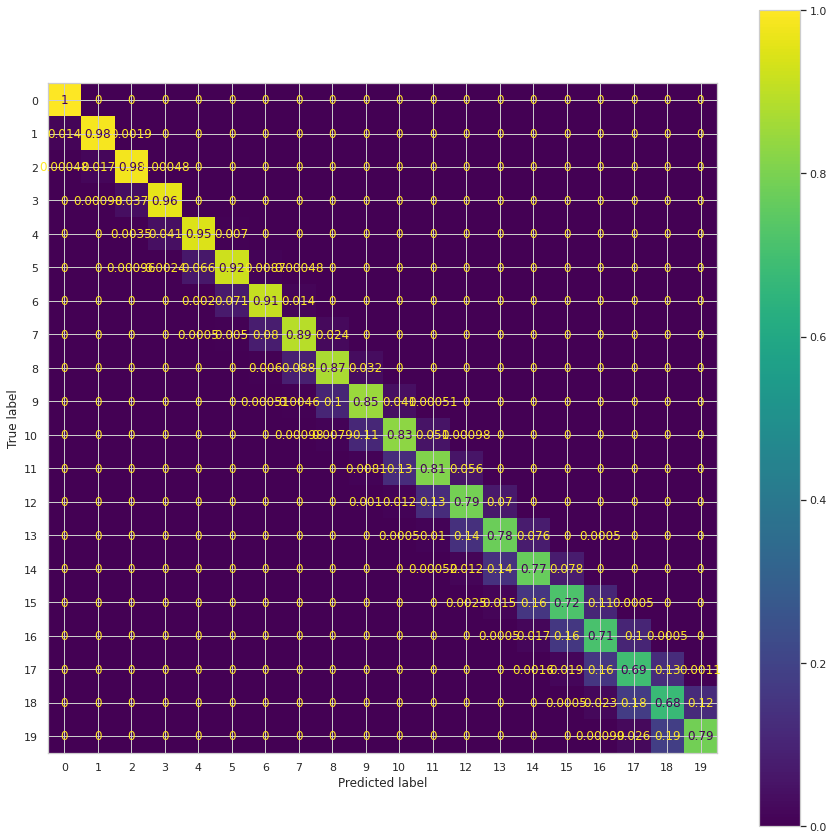


**Figure S2.** Confusion matrix showing the correspondence between predicted class and true class for machine-learned oligomer classification utilising a support vector machine (SVM).


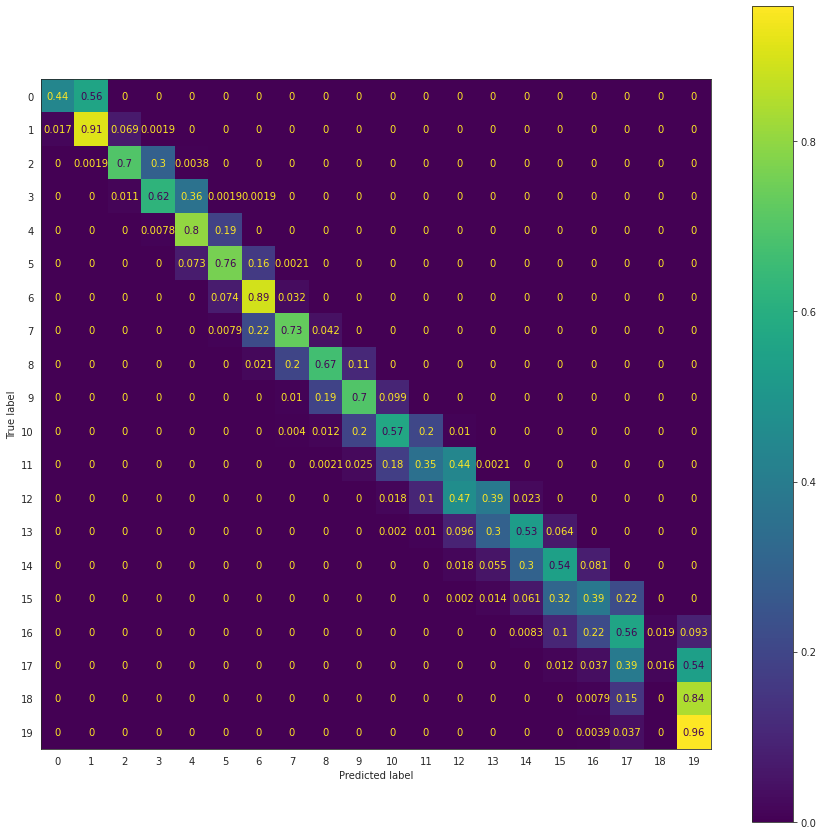


**Figure S3.** Confusion matrix showing performance of MLP model trained using raw photobleaching traces rather than a vector of specific features.
